# Uncertainty Visualization: Concepts, Methods, and Applications in Biological Data Visualization

**DOI:** 10.3389/fbinf.2022.793819

**Published:** 2022-02-17

**Authors:** Daniel Weiskopf

**Affiliations:** Visualization Research Center (VISUS), University of Stuttgart, Stuttgart, Germany

**Keywords:** visualization, uncertainty, layout, visual mapping, sampling, graph visualization

## Abstract

This paper provides an overview of uncertainty visualization in general, along with specific examples of applications in bioinformatics. Starting from a processing and interaction pipeline of visualization, components are discussed that are relevant for handling and visualizing uncertainty introduced with the original data and at later stages in the pipeline, which shows the importance of making the stages of the pipeline aware of uncertainty and allowing them to propagate uncertainty. We detail concepts and methods for visual mappings of uncertainty, distinguishing between explicit and implict representations of distributions, different ways to show summary statistics, and combined or hybrid visualizations. The basic concepts are illustrated for several examples of graph visualization under uncertainty. Finally, this review paper discusses implications for the visualization of biological data and future research directions.

## 1 Introduction

Data uncertainty can seriously affect its analysis and subsequent decision-making. Therefore, uncertainty should be considered in the context of visual data analysis and communication. This is well understood in many disciplines that deal with measured data. For example, error bars are widely used to indicate the uncertainty that comes with measurements, indicating standard mean of error or related descriptions of variability or uncertainty. However, uncertainty is not restricted to measurements but can also originate from numerical error in simulations, uncertainty in devising models, or many other sources.

In this paper, we discuss approaches to uncertainty visualization that do not restrict themselves to error bars. We address the problem of uncertainty visualization from a broader perspective, going beyond traditional statistical graphics and supporting more complex data than individual univariate distributions of data values, and therefore, linking to advanced visualization techniques. For many reasons, uncertainty visualization is difficult and considered one of the top research problems in visualization (Johnson, 2004). We will discuss some of the reasons and show strategies to address the problems.

There are already a number of survey papers on uncertainty visualization (*see* Section 2). We aim to complement them by adding some new perspectives: *1*) We focus on presenting general concepts of uncertainty visualization, with an emphasis on strategies for visual mappings. Here, we will use a categorization that partially differs from existing ones, focusing on structuring the design space. *2*) We build a bridge between sampling for visualizing uncertainty and modeling probability distributions, emphasizing the need for appropriate layout methods. *3*) The general concepts are illustrated with examples in biological data visualization, and implications for visualization in bioinformatics are discussed.

This paper is written from the perspective of visualization research, as for example, presented in conferences like *IEEE VIS*, *EuroVis*, or *IEEE PacificVis* and journals like *IEEE Transactions on Visualization and Computer Graphics* or *Computer Graphics Forum*. Therefore, we want to build a connection between visualization research in general and applications in bioinformatics. Although this paper has some characteristics of a survey, it is not meant to be a systematic survey of (biological) uncertainty visualization techniques. Instead, we often use examples from our own previous work to illustrate concepts. The main goal is broad coverage of principles, concepts, and approaches.

We see the following benefits: This paper provides an overview of general strategies that can be useful to visualize uncertainty in biological data. We also discuss practical aspects of integration into biological data analysis and visual communication, as well as future directions.

This paper is based on and extends a talk from VIZBI 2021.^1^

## 2 Related Work

There are many survey papers on uncertainty visualization that cover the topic from different perspectives. The seminal paper by Pang et al. (1997) adopts a general classification of visualization techniques and applies it to uncertainty visualization. Their classification is based on: the value of the input data and its corresponding value uncertainty; the position of the data within the domain, along with its positional uncertainty; the extent of location and value; the visualization extent (discrete vs. continuous); and axes mappings. This kind of classification or variants thereof are good because they bring order into the large collection of visualization techniques in general, and uncertainty visualization techniques in particular. They also facilitate choosing a visualization based on data characteristics. However, this taxonomy is less suited to understand how uncertainty visualization works and how we can use the design space to come up with new uncertainty visualizations. Therefore, Pang et al. (1997) also characterize uncertainty visualization techniques according to the following categories: adding glyphs, adding geometry, modifying geometry, modifying attributes, animation, sonification, and psychovisual approaches.

Griethe and Schumann (2006) base their survey on categories that can be associated with the visualization design space, similar to Pang et al.’s latter characterization: using free graphical variables, including additional graphical objects, animation, interaction, or leveraging other human senses. Later papers by Potter et al. (2011) and Brodlie et al. (2012) primarily structure their surveys according to data type, in particular, the dimensionality of the domain and the attached data values and uncertainties. Bonneau et al. (2014) organize their survey according to traditional representations (in 1D, 2D, and for probability density functions), visual comparison techniques, modification of attributes, glyphs, and image discontinuity. Ristovski et al. (2014) present a taxonomy focused on types of uncertainty and corresponding visualization challenges, concentrating on medical visualization. Siddiqui et al. (2021) summarize uncertainty visualization techniques for diffusion tensor imaging (DTI), considering the whole DTI visualization pipeline.

The above survey papers not only report on existing uncertainty visualization techniques, but also provide some background information: for example, on modeling uncertainty, how uncertainty data is acquired, and how uncertainty can be included in visualization processes or the visualization pipeline.

Jena et al. (2020) use a categorization with respect to publication type, publication venue, application domain, target user, and evaluation type. Their survey paper is accompanied by a web page^2^ that can be queried and browsed according to the categorization and that comes with consistent descriptions and representative images for each visualization technique. Especially the thumbnail images facilitate quick browsing for potential solutions to uncertainty visualization problems.

Padilla et al. (2020) start from the design space of uncertainty visualization, distinguishing graphical annotations of distributional properties (showing intervals and ratios, or distributions), visual encodings of uncertainty, and hybrid approaches. They also summarize some theories for uncertainty visualization, bringing in a perspective from psychology.

A recent survey article is by Kamal et al. (2021). They use the following categories to structure uncertainty visualizations: geometry, attributes, animation, visual variables, graphical techniques, and glyphs. They also summarize the conceptual basis of uncertainty visualization, sources and models of uncertainty, evaluation approaches, and future research directions.

As pointed out by Griethe and Schumann (2006), not all taxonomies are necessarily useful in structuring existing uncertainty visualizations because they might result in very uneven distributions of papers to categories. Therefore, our categorization of visual mappings is inspired by the design-space-oriented classifications from Pang et al. (1997), Griethe and Schumann (2006), Bonneau et al. (2014), Padilla et al. (2020), and Kamal et al. (2021). Our structure of visual mappings in Section 4 synthesizes a categorization based on variants from the above previous work, targeting strategies that can be used to develop new uncertainty visualization techniques.

The above survey papers are primarily based in the visualization research community. It should be noted that there is relevant related research in other fields as well. One prominent example is geography, geospatial science, and cartography; *see* the survey by MacEachren et al. (2005).

Related to perceptual and cognitive theories, Zuk and Carpendale (2006) applied principles by Bertin, Tufte, and Ware to examples of uncertainty visualizations to illustrate and better understand these and assess them. While theirs is not a survey paper, it provides a theoretical underpinning that is useful in understanding uncertainty visualization. The survey paper by Hullman et al. (2019) focuses on one aspect of uncertainty visualization: its evaluation. Examples of evaluation papers include the ones by Deitrick and Edsall (2006) or Sanyal et al. (2009), but many more are reviewed by Hullman et al.

Skeels et al. (2010) pick out another important aspect: what are relevant models and types of uncertainty for visualization? Furthermore, visualization in general has to consider the analysis tasks that should be supported. Murray et al. (2017) provide a task taxonomy for the analysis of biological pathway data that includes identifying uncertainty. Also in the context of bioinformatics, Hamada (2014) summarizes several approaches to handle uncertainty, in particular, recommending visual representations.

It should also be noted that there are other concepts that are related to uncertainty and have some overlap. For example, ensemble visualization aims to show members from an ensemble, which can be viewed as a special case of describing variability. Therefore, uncertainty and ensemble visualization techniques show substantial overlap. Wang et al. (2019) provide a survey of ensemble visualizations. Other related concepts comprise human trust building or data provenance, as integrated into the framework by Sacha et al. (2016).

Some of the example visualizations that we demonstrate in this paper are based on (joint) research that went into the doctoral theses by Görtler (2021) and Schulz (2021). These theses also provide overviews on quantification for uncertainty visualization and approaches to making visualizations aware of uncertainty. In particular, they discuss sampling and layout methods for uncertainty visualization.

In summary, we do not want to replace the aforementioned surveys that come with a broad coverage of previous literature. Instead, our goal is to provide some additional perspective on the problem of uncertainty visualization. In contrast to most of the previous survey papers, we use many examples from biological data visualization to illustrate uncertainty visualization. Furthermore, we present a slightly different categorization of visual mappings and point out specific issues that were not the focus of previous papers: the role and challenges of sampling for the implicit visualization of distributions, and the relevance of layouts for advanced uncertainty visualization.

## 3 Overview of Uncertainty Visualization

**FIGURE 1:**
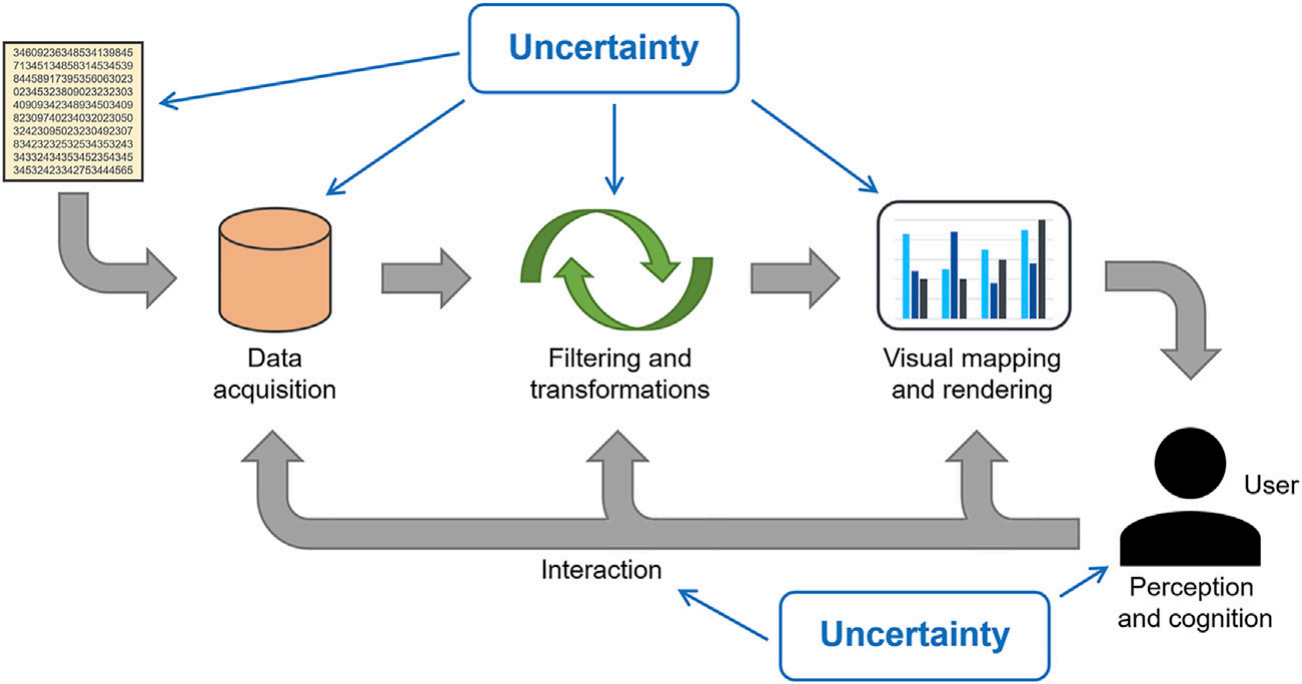
Visualization pipeline including uncertainty.

This section provides an overview of where and how uncertainty plays a role in visualization. We use the visualization pipeline to organize and structure the effects of uncertainty, *see* Figure 1.

Many of the previous survey papers employ the visualization pipeline as well (Pang et al., 1997; Griethe and Schumann, 2006; Brodlie et al., 2012; Ristovski et al., 2014; Kamal et al., 2021; Siddiqui et al., 2021). Our description is based on a pipeline for scientific visualization by Haber and McNabb (1990) and the related one for information visualization by Chi and Riedl (1998). However, we extend it slightly by including the human user (with their perceptual and cognitive aspects) and the interaction of the user with different stages of the pipeline. All of these need to consider uncertainty as well.

Following Brodlie et al. (2012), we can distinguish between *visualization of uncertainty* and *uncertainty of visualization*. The former is the typical focus when we address uncertainty visualization: showing the uncertainty that comes with the data. The latter term describes the additional uncertainty introduced by visualization—on top of the uncertainty associated with the data. Often, these two terms are treated in a combined fashion because they form the overall uncertainty in the final visualization.

There is an important point that comes with the visualization pipeline: The different stages have to be made uncertainty-aware and they have to be able to propagate uncertainty through the pipeline.

### 3.1 Uncertainty Modeling and Acquisition

One difficulty is that the term *uncertainty* is not well defined in the field of uncertainty visualization. In particular, there is not a unique model of uncertainty. In some vagueness, it may refer to error, variability, or other aspects that may degrade the quality of data and visualization. Therefore, a typical challenge in using uncertainty visualization is to first understand the type of uncertainty that is to be shown. This is one of the critical elements in linking visualization to the specific application at hand.

There are a number of different taxonomies to describe various types of uncertainty. For example, we can distinguish between accuracy/error, precision, completeness, consistency, lineage, currency, credibility, subjectivity, and interrelatedness (MacEachren et al., 2005, 2012). Skeels et al. (2010) provide a classification in the form of measurement precision, completeness (covering missing values, sampling, aggregation), inferences (covering predictions, modeling, and descriptions of past events), disagreement, and credibility.

These models of uncertainty are determined by the sources of uncertainty and how it is used in the visualization and analysis. For example, there might be measurement errors, numerical errors from simulations, missing or corrupted data, variability from statistical observations, or from aggregating larger chunks of data into a compressed form.

Despite this vagueness, many uncertainty visualization techniques are based on some kind of probabilistic modeling of data uncertainty, i.e., in the form of probabilities or probability density functions. Furthermore, such uncertainty is often acquired by aggregation or computing summary statistics such as mean, median, standard error, percentiles, etc. Therefore, unless stated otherwise, we assume such probabilistic modeling and that uncertainty is described by summary statistics, by parameters of probability models (like parameters of probability density functions), or by providing original data samples (from which statistical descriptions could be computed).

### 3.2 Filtering and Transformations

Usually, the input data is not directly mapped to a visual representation. In particular, for large or complex data, it might be necessary to reduce the amount of data shown. Therefore, filtering and transformations of the input data are required to obtain data that is more informative: it might be reduced in amount or complexity, or important features might be extracted for highlighting. Therefore, this stage of the visualization pipeline is critical for avoiding or reducing information overload.

Filtering can be as simple as selecting data items based on allowed ranges of data, which might be specified by the user or driven by the distribution of the input data. Clustering is a common transformation approach in visualization because it facilitates structuring and grouping data, supporting summarized and compact representations; *see*, for example the survey paper by Xu and Wunsch (2005). Another typical example is the use of dimensionality reduction methods (or multidimensional projection) that allow one to transform high-dimensional input data to 2D or 3D data, leading to an easy mapping to visualization space. For background reading, *see*, for example, the book on nonlinear dimensionality reduction by Lee and Verleysen (2007). Modeling in high-dimensional space is very generic and can be used for manifold applications. One bioinformatics example is the representation of phylogenetic trees that lends itself to multidimensional projection and uncertainty visualization (Willis and Bell, 2018).

Complex types of transformations can introduce additional uncertainty, i.e., they can lead to increasing visualization uncertainty. For example, multidimensional projections cannot fully guarantee the preservation of the original characteristics of the input data. The introduced distortions from projections can be identified and visualized, as summarized in a survey paper by Nonato and Aupetit (2019). Or, as in fuzzy clustering (Baraldi and Blonda, 1999), transformations might provide gradual or fuzzy assignments to clusters on purpose, again resulting in uncertainty that only originates at this stage of the visualization pipeline.

However, transformations do not only contribute to visualization uncertainty, they also have to be able to propagate incoming uncertainty downstream the pipeline. In this case, the transformation stage does not add errors during the process, but it has to pass them through appropriately. Since transformations can be highly nonlinear, this propagation might be hard to compute and it might distort the uncertainty substantially.

**FIGURE 2:**
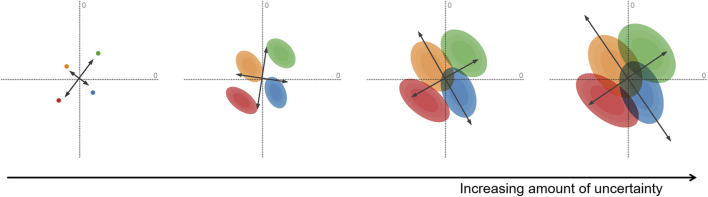
Uncertainty-aware PCA applied to a simple test data set with four points with two data dimensions. Increasing the amount of uncertainty attached to the data points, we obtain wider and wider distributions (here, normal distributions) that lead to larger and larger coverage in the visualization. However, the changes in the distributions of uncertain input even affect the computation of the PCA axes: they are rotated to reflect the changing distribution of the input. Image: ^©^ 2020 IEEE. Reprinted, with permission, from Görtler et al. (2020).

For example, uncertainty-aware principal component analysis (PCA) (Görtler et al., 2020) incorporates the uncertainty in high-dimensional data points to adapt the computation of the projection operator. Figure 2 illustrates the effect of uncertainty on PCA. Uncertainty not only affects the display of the data points (which get wider with increasing uncertainty), but it even impacts the projection directions as indicated by the rotation of the PCA axes.

This example demonstrates the importance of making transformations aware of uncertainty. While there are uncertainty-aware variants already for some of the typical filtering and transformation techniques, there is still much room for future work in this direction. This is a research question not just for visualization but any field where numerical analysis of uncertain data is performed. Therefore, related methods may be developed in a range of different research fields.

### 3.3 Mapping and Rendering

The mapping stage of the visualization pipeline takes the transformed data and produces a renderable representation, for example, in the form of geometry together with attributes like color or opacity. Such geometry could be the set of points to be shown in a scatterplot, or a triangle mesh for an isosurface. This representation is then rendered to generate the final visualization image. The actual rendering is mostly well understood, with manifold techniques available from computer graphics.

In contrast, the mapping stage is in the center of visualization because it is the critical link between data and image. Developing appropriate visual mappings can already be hard for visualization without uncertainty, and it becomes even more challenging for uncertainty visualization. Visual mapping is a focal point of this paper, with a detailed discussion of mapping strategies in a dedicated later section (*see* Section 4).

### 3.4 Perception and Cognition

Visualization only works in combination with a human that uses imagery to understand the data or communicate with others. Therefore, visual perception and cognition play a critical role in visualization in general (Ware, 2021). In this context, user-oriented evaluation of visualization techniques is relevant and challenging at the same time (Lam et al., 2012); there is even a specialized series of workshops addressing evaluation methods for visualization.^3^

Including uncertainty makes understanding and assessing perception and cognition even harder. In particular, we have to be careful in designing uncertainty visualization so that it is correctly understood by the recipient. For example, even researchers have problems understanding and correctly judging the information encoded in the, at first sight quite simple, visualizations in the form of confidence intervals and error bars (Belia et al., 2005). These findings led to recommending alternatives to error bars (Correll and Gleicher, 2014).

Error bars are quite simple and very common; therefore, it is conceivable that more complex uncertainty visualizations could be affected even more from difficulties with perceiving and understanding them (Boukhelifa and Duke, 2009). Assessing cognitive aspects is particularly hard when complex decision-making has to be done under uncertainty (Padilla et al., 2021). It can also make a difference whether experts or non-experts use and read uncertainty visualizations. For example, Tak et al. (2014) study how non-experts perceive and understand typical examples of uncertainty visualizations. Some theories and further examples of perceptual and cognitive considerations are summarized by Padilla et al. (2020). Similarly, special attention needs to be paid to perform a proper evaluation of uncertainty visualization; *see* the survey paper by Hullman et al. (2019).

### 3.5 Interaction

While uncertainty visualization sometimes targets passive consumption, for example, in the form of an illustration for visual communication, it is often employed in an interactive environment. Interactive visualization or visual analytics are typically used to facilitate visual data analysis.

**FIGURE 3:**
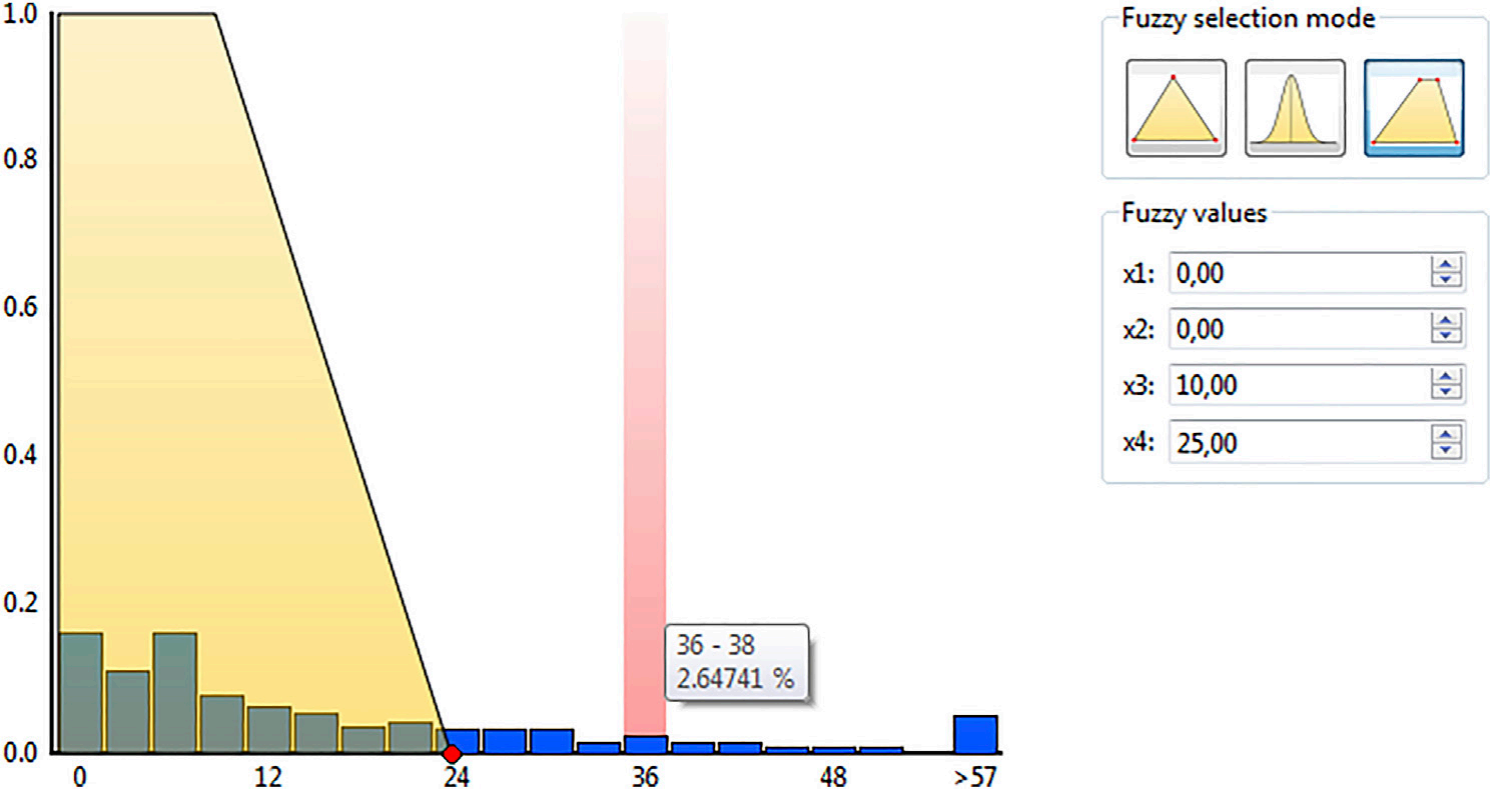
Interactive selection by fuzzy filtering. The data context is given by a histogram (blue), along with some data details (at the column highlighted in light red). The user chose a trapezoid function for fuzzy selection, setting the four parameters x1 to x4 accordingly. The yellow area indicates the selection. Image: “Speed filter” by Höferlin et al. (2011) licensed under CC BY 3.0.

Therefore, the interaction needs to be made aware of uncertainty as well. This includes how data serves as the basis for the interaction technique. However, uncertainty can also be present in the interaction itself. The user may not be sure about what they want to exactly specify with their input. For example, the input may serve as a threshold for interactive filtering. Here, uncertain input may be specified by sliders that are connected to uncertainty in the form of probability density functions (Greis et al., 2017). Another example is fuzzy selection facilitated by several selection modes, including triangle and trapezoidal shapes (Höferlin et al., 2011); *see* Figure 3.

Overall, the topic of uncertainty-aware interaction has not received much attention in visualization research. Therefore, we see the need for more work in this direction. One challenge is that this is directly linked to the difficult problem of understanding cognition and mental models of uncertainty—related to the previous subsection. Another challenge is that uncertainty-aware interaction has to be adapted to the different steps of the visualization pipeline. For example, specifying uncertain value ranges (as in the two examples above) is appropriate for defining value-oriented filtering, but different inputs are needed for other filters, transformations, or visual mappings.

### 3.6 Integration

So far, we have discussed the stages of the visualization pipeline one after another. However, uncertainty needs to be propagated through the whole process (Wu et al., 2012). Unfortunately, it can be hard to accurately compute uncertainty propagation because the various stages of the visualization pipeline can be quite complex and highly nonlinear. In particular, it is challenging to include human perception, cognition, and interaction in this propagation. Another problem is that typical uncertainty propagation methods tend to increase uncertainty substantially, especially, when transformations are highly sensitive or when there is a sequence of transformations. The uncertainty estimates are often too conservative and, therefore, unrealistically large if uncertainty is passed on without a full model of the data and visualization process. By including additional information, more accurate and tighter descriptions of uncertainty might be possible.

Overall, the whole visualization process should be made aware of uncertainty (Correa et al., 2009). Since this might not be fully possible, we recommended assessing the visualization workflow and identifying the most substantial contributors to uncertainty, along with the intended visualization goals and tasks. Based on this, efforts in incorporating uncertainty can be directed to the most relevant components.

## 4 Visual Mapping

**FIGURE 4:**
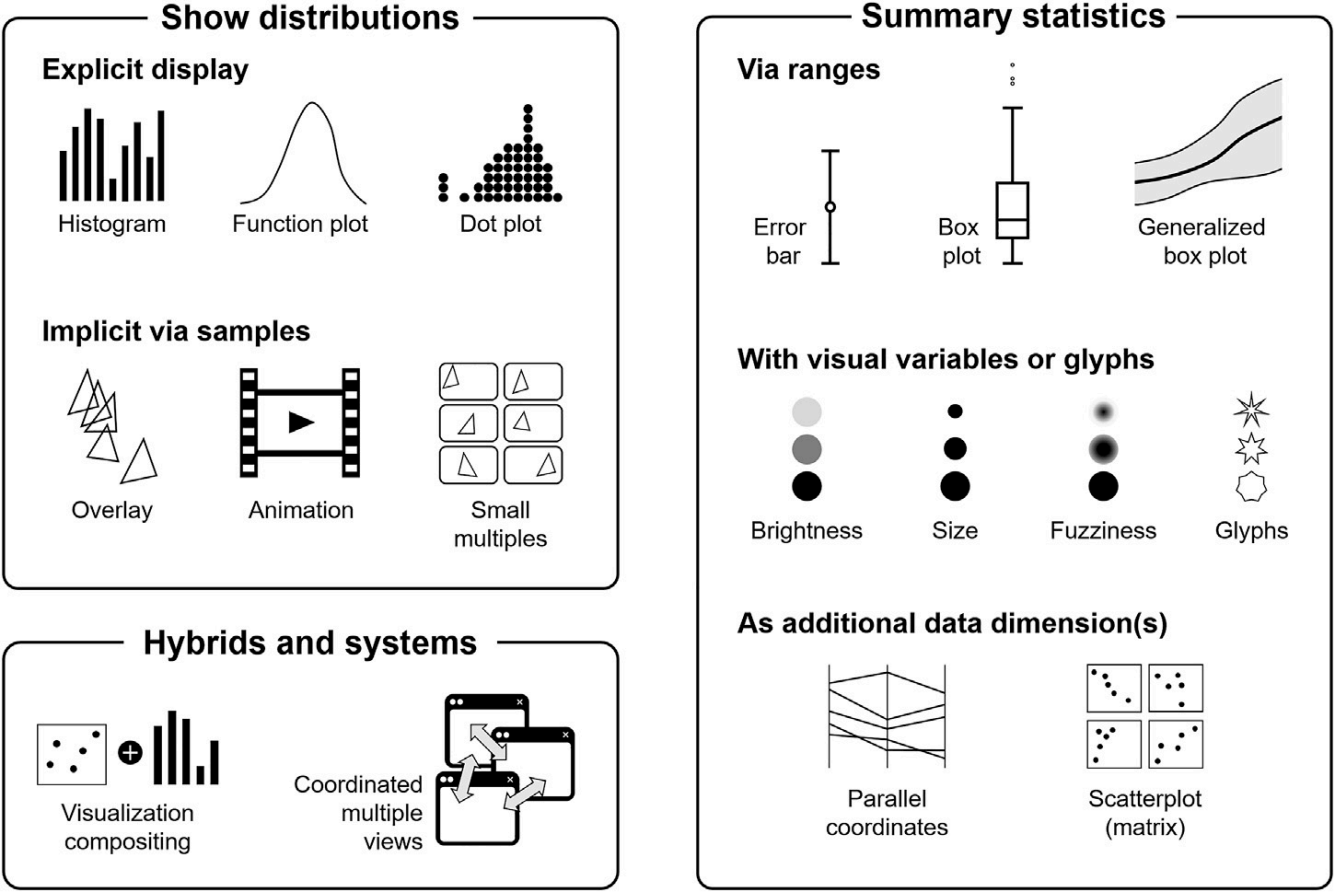
Overview of visual mapping strategies to show uncertainty. The individual visualization techniques only serve as illustrative examples and are not meant to provide a complete list of methods.

In this section, we discuss visual mappings of uncertainty in more depth. Visual mapping strategies are summarized in Figure 4. This figure is inspired by the visual summary used by Padilla et al. (2020). However, our categorization partially differs from theirs and also from the other taxonomies reviewed in Section 2. Please note that the icons in Figure 4 illustrate typical representatives for the respective strategy, but they are not meant to be comprehensive, i.e., it is to be understood that there are more visualization approaches for the respective strategy.

If not stated otherwise, we assume a probabilistic model of uncertainty—typically in the form of probability density functions (PDFs) describing distributions of data values. These may be reduced to concise characteristic descriptions, for example, by summary statistics. Or the raw samples might be available before computing summary statistics or constructing PDFs.

### 4.1 Explicit Visualization of Distributions

Let us start with the first kind of visual mappings: these aim to show distributions explicitly and fully. For example, a PDF can be seen just as a function and, therefore, a function plot displays the uncertainty distribution comprehensively. If the uncertainty data is provided as “raw” sampled data, traditional histograms in the form of bar charts can be employed. An alternative is the dot plot (Wilkinson, 1999), or the nonlinear dot plot (Rodrigues and Weiskopf, 2018) for higher dynamic range. The sample-based visualization can even be used if only a PDF is available: just by drawing samples from the given PDF.

The advantage of the explicit visualization of distributions is that they provide full disclosure of uncertainty information. A disadvantage is the extra visualization space needed: frequency or probability (density) are plotted along an axis (usually, the vertical axis) that is perpendicular to the axis that carries the data values (usually, the horizontal axis), i.e., we require 2D space instead of 1D space just for the data axis.

A related characteristic is that the 2D visualization axes carry different meanings: data values vs frequency or probability (density). This difference can have benefits if we want to clearly separate the two meanings. At the same time, it can lead to problems if the visualization space is taken as one 2D space.

Overall, the explicit visualization of distributions is typically employed for rather small data sets, or for data drill down to show detailed views on large data sets.

### 4.2 Implicit Visualization of Distributions *Via* Samples

Some problems of the above explicit visualization can be addressed by showing distributions implicitly *via* samples drawn from the distribution. The basic process is as follows: In the first step, the distribution is sampled to produce potential realizations of the data, compatible with the uncertainty representation. Each sample is treated as if it was not affected by uncertainty. In the second step, each sample is visualized. The last step is responsible for showing the visualizations of all samples in some combined fashion.

Variants of this uncertainty visualization approach mostly differ in the way they implement the last step. One option is to overlay or composite the individual visualizations of the samples, for example, by additive blending or alpha blending (Schulz et al., 2017). Another option is the use of animation, showing individual visualizations one after another, e.g., in the form of the animation of potential realizations (here, surfaces) by Ehlschlaeger et al. (1997) or in the form of Hypothetical Outcome Plots (Kale et al., 2019). Yet another option places individual visualizations next to each other in one large image, in the form of small multiples (Tufte, 1990).

All of these implicit visualizations have the advantage that they just use the regular visualization space, i.e., there is no need for extra space with other semantics, as for the explicit visualization of distributions. Therefore, the uncertainty visualization should be understandable by the user if they are familiar with the original, non-uncertainty-affected visualization. The variants for the last step have specific advantages and disadvantages. The overlay approach has the advantage that it essentially needs just the visualization space that a single visualization would need. Another advantage is that it results in a static image, i.e., it can be flexibly used in visual communication, and it gives the user enough time to carefully inspect the visualization. The main disadvantages are overplotting, clutter, and ambiguities that can arise from compositing many visualization samples.

The animation approach avoids this overplotting and provides some advantages in interpreting uncertainty (Kale et al., 2019). However, this approach comes with typical problems of animated visualization that can be difficult for analysis tasks (Robertson et al., 2008). Animation also has some issues with scalability with the number of samples shown: it is hard to get a quick overview, which in contrast is possible with the single and static image in the overlay approach.

Small multiples are similar to the animated display because they show individual visualizations independently. The main difference is that animation puts the individual images one after another along time, whereas small multiples place them next to each other in an enlarged visualization space. Similarly to animation, this approach avoids overplotting. However, it needs much visual space and, again, has issues with the scalability regarding the number of samples. Also, it might be hard to perceive and interpret differences between the individual visualizations.

While the visual representation is quite different in the three approaches, they all share the need for appropriate registration or alignment between the individual images—whether these are the images that go into the blending, animation, or as part of the small multiples. The potential problem is that individual images may look very different even if the sampling from the distribution leads to similar data. In other words, some visualization techniques can be very sensitive to slight changes in the input data. For example, many graph drawing algorithms can lead to quite different outputs even if the input is similar (e.g., in the form of rotated images). Section 5 discusses the registration problem and visualization approaches for the example of graph drawing in more detail.

### 4.3 Summary Statistics as Range Plots

The above explicit and implicit visualizations aim to show the full characteristics of the underlying distributions. However, it is often sufficient to convey just some aggregated or concise representation of the distributions. For example, summary statistics may rely on some indicator of central tendency (such as mean or median) and variability (like standard deviation, standard error, or percentiles). Statistical graphics then maps these summarizations to visual representations such as error bars or box plots.

From the perspective of visualization, these mappings lead to a representation of ranges. For example, a typical box plot shows the range from the 25 percentile to the median and then to the 75 percentile, where each of the boundaries is indicated by a line in the box plot. Another observation is that these range plots need additional visualization space to make room to show the ranges. Therefore, they work fine for traditional statistical plots where one has just a few data items that are enriched by statistical graphics. However, it becomes harder to fit the range plots into a visualization that already needs a lot of space on the image to show data without uncertainty.

**FIGURE 5:**
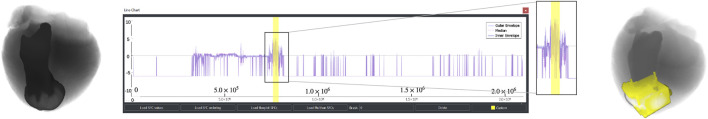
Functional box plots applied to a data-adaptive space-filling curve. The volume rendering on the left shows the median of a heart ischemia simulation. The center part shows the variability in the input along the horizontal axis that corresponds to a space-filling curve cutting through the volume. Here, the user can select (brush) ranges of interest such as an area where the potential value is larger than 3 eV. The corresponding ischemic region is highlighted (yellow) in the volume rendering on the right. Image: ^©^ 2021 IEEE. Reprinted, with permission, from Zhou et al. (2021).

One strategy maps the original data to a lower-dimensional visual representation that supports adding ranges. For example, 3D volume data can first be reduced to a 1D curve by letting a space-filling curve cut through the volume; afterward, we can apply bands or range representations around the curve (Demir et al., 2014). Figure 5 shows an example that uses a data-adaptive space-filling curve to perform the reduction to 1D (Zhou et al., 2021). Here, the data comes from a heart ischemia simulation; *see* Rosen et al. (2016) for background reading.

Another strategy relies on a generalization of the idea of a box plot, utilizing the concept of statistical depth, which can be seen as the generalization of medians or percentiles in complex data. For example, contour box plots indicate parts or ranges in a spatial domain that correspond to certain values or ranges of depth (Whitaker et al., 2013). Another example shows variability in functions by function box plots (Mirzargar et al., 2014).

Yet another strategy places small glyphs on the domain to indicate data ranges at respective locations. For example, radial glyphs can be used to represent the range of vector quantities at respective locations in a vector field (Hlawatsch et al., 2011). Furthermore, the concept of displaying ranges can be extended to rather complex geometric representations, for example, in order to visualize confidence intervals for fiber tracking for showing 3D brain structures (Brecheisen et al., 2013).

In general, range plots provide a representation of summarizing characteristics of uncertainty and are rooted in well-known visual representations from statistical graphics. Therefore, they can be used without much learning required by recipients of the visualization. Another advantage is that ranges show quantitative information about summary statistics. However, there is a caveat: as mentioned before, even traditional error bars might be misinterpreted (Belia et al., 2005). Furthermore, the principle of showing distinct ranges can lead to the wrong interpretations because they might lead to introducing false categorical boundaries, e.g., inside vs outside regions (Padilla et al., 2020). Finally, range-based visualizations tend to need substantial extra space on the visualization image that might not be available.

### 4.4 Summary Statistics in Visual Variables and Glyphs

We can still use characteristic quantities from summary statistics, but now map them to visual channels, such as color, brightness, texture characteristics, etc. There are many different design choices for this mapping, with different characteristics and effectiveness for uncertainty visualization. Most of these mappings focus on including the variability of the input data into the visual representation.

For example, MacEachren et al. (2012) link visual channels for uncertainty representation to the semiology of graphics by Bertin (1983). Visual variables (also called retinal variables by Bertin) describe a set of visual primitives from which we can construct a visualization. MacEachren et al. (2012) investigate the following visual variables according to their usefulness for uncertainty visualization in terms of intuitiveness and task performance (focusing on map reading): location, size, color hue, color value, color saturation, orientation, grain, arrangement, shape, fuzziness, and transparency. These exhibit different adeptness for uncertainty visualization, for example, fuzziness shows a high level of intuitiveness in their study.

These visual variables are only one approach to structure the design space. Boukhelifa et al. (2012) provide a grouping into three main categories: color-oriented approaches (hue, saturation, or brightness), focus-based methods (mapping uncertainty to contour crispness, transparency, or resolution), and geometric mapping (e.g., sketchiness in rendering, distorting line marks). Animation (for example, oscillating displays) can also be used to represent uncertainty (Pang et al., 1997).

In particular, if such mappings are used to modify larger graphical elements such as icons or glyphs, we have a quite large design space that allows us to represent uncertainty. For example, Vehlow et al. (2013) modify attributes of (larger) nodes to show uncertainty: by color gradients or alternatively by star-shaped icons. In another application, glyphs are designed to represent the distribution of fibers (Schultz et al., 2013).

**FIGURE 6:**
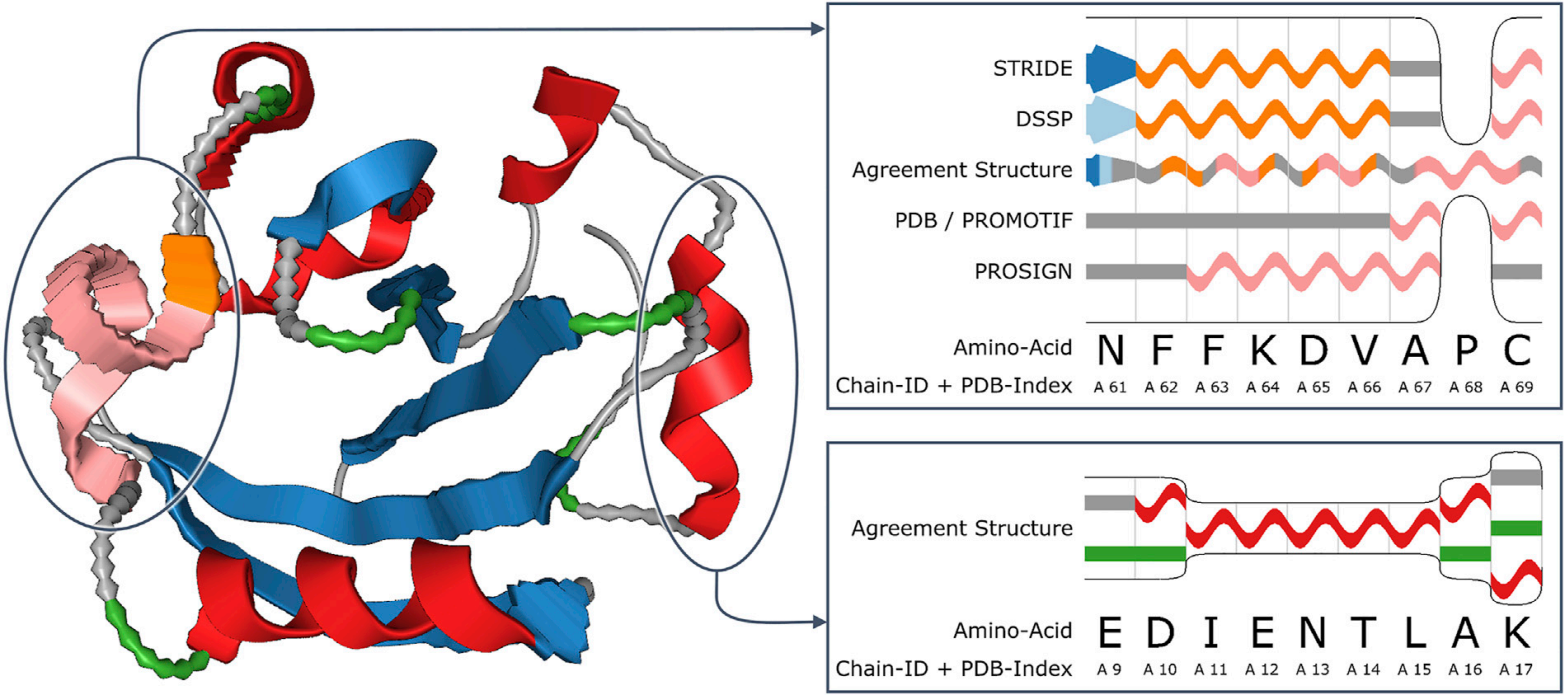
Visualization of uncertainty in secondary structure assignment for the example of the photoactive yellow protein of *E. coli* (PDB ID: 2ZOI). The ribbon diagram (left image) represents this uncertainty in the form of waviness in the geometric shape. The insets show details of parts of the sequence diagrams (images to the right), stacking the differing assignments along the vertical axis. The agreement structure merges the assignment results where possible. Image: ^©^ 2018 IEEE. Reprinted, with permission, from Schulz et al. (2018).

Figure 6 shows an example of a 3D visualization using waviness to represent uncertainty, here for the uncertainty that comes from disagreement in secondary structure assignments (Schulz et al., 2018). Alternative visualization methods for the uncertainty in secondary structure assignments are discussed by Hamada (2014). Another example of uncertainty visualization for proteins is by Maack et al. (2021), who address the visual representation of uncertainty in the conformation of proteins.

Uncertainty encoding in visual variables has the advantage that it can, if done appropriately, provide an intuitive visualization of uncertainty that integrates well in existing non-uncertainty visualization techniques because the original visualization technique might not be changed substantially. However, these visualization techniques tend to focus on rather qualitative representations; it is usually hard to read off accurate uncertainty information. Another issue is that there can be conflicts in choosing the visual variables: one has to balance between the need for a good visual representation of uncertainty and the other kinds of information that should be shown in the visualization. Also, one has to be careful that there might be (negative) interactions between visual variables that can make it hard to include uncertainty information in an existing visualization.

In summary, this mapping approach needs careful design but can lead to good qualitative overview visualizations.

### 4.5 Uncertainty as Additional Data Dimension

The above approaches to including summary statistics essentially use different visual mappings to integrate the additional information that comes with summary statistics. To this end, they employ different variants of visual mappings.

However, we can also cast the problem of uncertainty visualization into the problem of multivariate visualization. For example, let us consider the case of data with *n* data attributes or dimensions. And let us assume that each data dimension comes with uncertainty described by one measure of variability (e.g., standard error of means). Then, we just increase the dimensionality of the data from *n* to 2*n* to represent, for example, both the means and the standard error of means. From this perspective, we have transformed the problem of *n*-D visualization (for precise data) to the problem of 2*n*-D visualization (for uncertain data). Therefore, we can apply standard visualization techniques that can deal with multiple data dimensions (Wong and Bergeron, 1994), such as parallel coordinates (Inselberg, 1985; Heinrich and Weiskopf, 2013) or scatterplot matrices.

The advantage of this approach is that it can readily use existing visualization techniques and, thus, there is no or only little extra effort required. Another advantage is that many of these visualization techniques support accurate visualization. For example, parallel coordinates or scatterplots let us read off quantitative information accurately from the diagrams, which is in contrast to the more qualitative visualizations in the previous subsection. The important disadvantage is that we lose the nature of uncertainty in the visualization: there is no intuitive connection to variability. Therefore, this approach is less useful for conveying uncertainty in visual communication, and it can be prone to misinterpretations even by expert analysts.

### 4.6 Hybrid Visualizations and Systems

The above visualization techniques can be used in combination or together with other non-uncertainty visualizations, leading to hybrid visualizations. One strategy is to build a composition of a larger visualization that combines different visual representations. Often, the uncertainty visualization is placed next to the usual, non-uncertainty visualization. For example, Holzhüter et al. (2012) use an explicit representation of uncertainty with additional bar charts placed next to the actual visualization to show uncertainty from the visualization of biological expression data. Typical strategies use juxtaposition of visualizations or overlays to perform the composition. The summary plot (Potter et al., 2010), for example, integrates a box plot, histogram, a display of statistical moments, and a plot of the distribution.

Another common strategy employs multiple coordinated views (Baldonado et al., 2000) to link separate visualization views, often in connection with brushing and linking (Becker and Cleveland, 1987). Multiple coordinated views are popular in larger visualization or visual analytics systems because they allow us to represent data from different angles.

Hybrid visualizations, in particular, multiple coordinated views, are quite common and useful for uncertainty visualization because they allow us to reduce the complexity of each individual visualization, which is especially important for the increased difficulty that comes with including uncertainty in the visualization. However, we have to be careful that we do not overload the user with too complex combinations and hard-to-handle interactions. Therefore, attention needs to be paid to an appropriate design of the visualization and interaction.

## 5 Example: Graph Visualization

We want to illustrate the aforementioned concepts for the example of graph visualization, with a focus on node-link diagrams. There are several reasons for choosing this example: *1*) It is a rather complex kind of visualization already for the traditional non-uncertainty case. Therefore, it serves to show what challenges and opportunities arise with advanced uncertainty visualization. *2*) It is an example of visualization of abstract data (often referred to as information visualization), which is less well explored than uncertainty visualization for scalar or tensor fields (as in scientific visualization). Therefore, this example illustrates the current developments in uncertainty visualization. *3*) Graphs are a versatile form of data representation with manifold uses in bioinformatics and beyond. Therefore, there is direct relevance for applications in biological data visualization.

Graph visualization is a large subfield of visualization, with many techniques available; *see*, for example, Battista et al. (1998), von Landesberger et al. (2011), and Beck et al. (2017) for background information.

Our first example (Vehlow et al., 2012) aims at the visualization of biochemical reaction networks. Such networks play a role in understanding certain cell functions or diseases. Our first step is to interface with the underlying modeling of the system and data acquisition (the early steps of the visualization pipeline; *see* Section 3). In this example, the modeling is circled around ordinary differential equations (ODEs) that are connected in the form of a directed graph. Vertices of the graph represent species and edges correspond to reactions. Besides regular edges, there might be hyper-edges representing regulatory interactions. Uncertainty is introduced by noise in measurements and, subsequently, by the uncertainty that comes with Bayesian parameter estimation.

**FIGURE 7:**
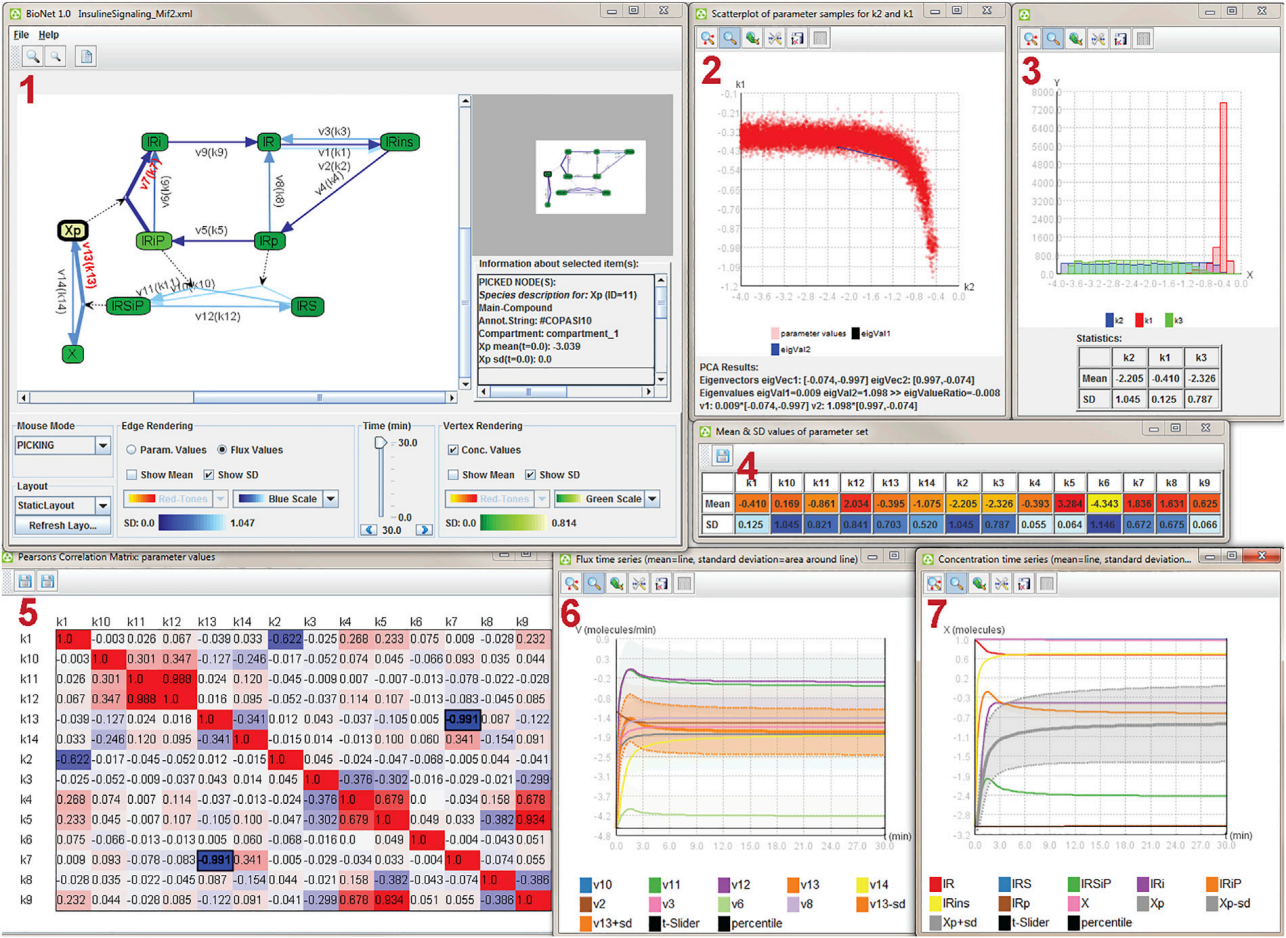
Coordinated multiple views for uncertainty visualization, analyzing an insulin signaling model. Image: ^©^ 2012 IEEE. Reprinted, with permission, from Vehlow et al. (2012).

From the visualization perspective, we are dealing with data in the form of a graph with uncertain and time-dependent attributes on the graph’s vertices and edges, where time dependency comes from the temporal evolution of the reactions. Figure 7 shows a snapshot from a visualization system that facilitates the uncertainty-aware visual analysis of such kind of data. It takes the general approach of multiple coordinated views with brushing-and-linking (Section 4.6) to present the data from different angles and with different levels of detail. The node-link graph visualization (Figure 7 (1)) shows the topological structure of the graph and includes the visualization of uncertainty for edge and vertex attributes *via* color-coding of respective standard deviations; therefore, the uncertainty visualization uses a visual variable (here, color) to represent summary statistics (here, standard deviation); *see* Section 4.4. The same color-coding is used to show uncertainty in a detail view (Figure 7 (4)).

The visualization system also includes bands around temporal function plots (Figure 7 (6), (7)), implementing a range visualization of summary statistics; *see* Section 4.3. Furthermore, there is an explicit display of value distributions in the form of histograms (Figure 7 (3)), again focusing on selected details; *see* Section 4.1. Value distributions are also shown in an overlay of sample points in a scatterplot (Figure 7 (2)); *see* Section 4.2. Finally, there is additional data processing and extraction of information that is aligned with uncertainty-affected input: fitting of axes due to principal component analysis (Figure 7 (2)) and correlation according to Pearson coefficients (Figure 7 (5)).

This example demonstrates that multiple different perspectives are often required to obtain a comprehensive view and analysis of uncertain data. The different views are also needed to support a variety of analysis tasks. In this example, the system was developed and evaluated in collaboration with domain experts.

The next example shows uncertainty visualization for the case where uncertainty is introduced not at the data acquisition stage, but only later during the visualization pipeline in the transformation stage (Vehlow et al., 2013). Here, graph clustering (i.e., community detection) is applied to facilitate data analysis of a protein–protein interaction network on different levels of granularity: graph nodes are combined in groups that can be then shown by meta-nodes representing groups of nodes. Uncertainty is introduced by applying fuzzy clustering, which can lead to the gradual membership of a node in several groups.

**FIGURE 8:**
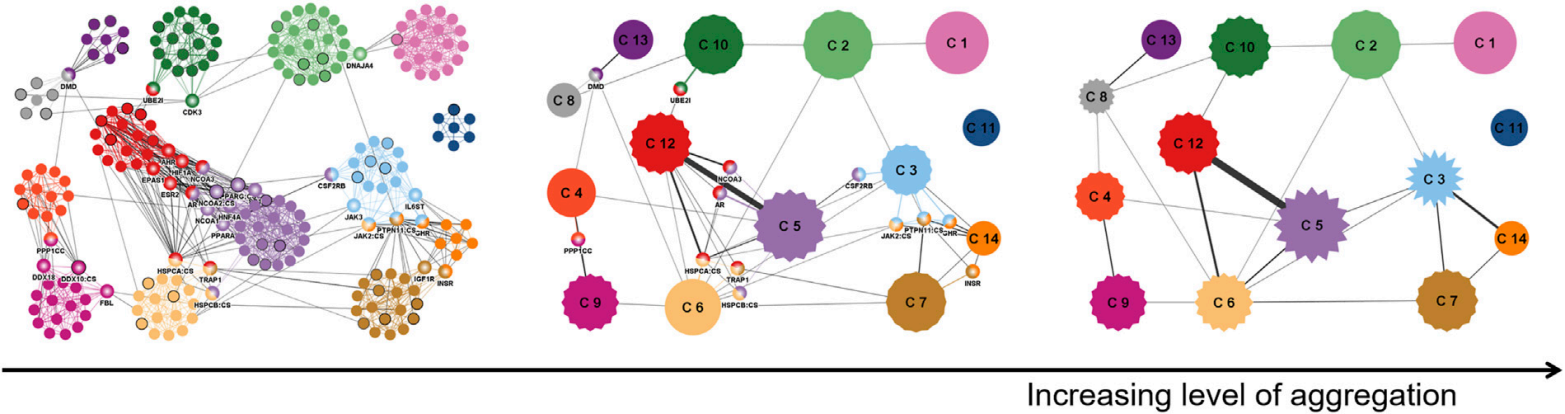
Visualization of data of a protein–protein interaction network (Jonsson and Bates, 2006). The images show the same subnetwork as in the original article, containing 1,253 weighted interactions between 232 proteins, where edge weights indicate confidence scores for the interactions. From left to right: increasing level of aggregation, starting from the original network data. Image: ^©^ 2013 IEEE. Reprinted, with permission, from Vehlow et al. (2013).

In this example, the amount of uncertainty associated with grouping in a meta-node is represented by the amplitude of spikes in star-shaped icons (*see* the middle and right image in Figure 8), i.e., summary statistics is represented in a visual variable of the icon. In addition, original nodes may belong to several fuzzy clusters; here, the certainty of membership is shown again by a visual variable, now in the form of a color gradient within a node (several examples in the left image in Figure 8). Besides the visual mapping to visual variables, the layout of the network has to incorporate the information from fuzzy clustering, i.e., the mapping stage of the visualization pipeline has to be aware of the uncertainty model.

The previous two examples have focused the graph visualization aspect on showing summary statistics *via* visual variables. Our third example shifts the focus: how does uncertainty in edge attributes affect the geometry of the node-link diagram? The uncertainty model assumes distributions of weights on edges. Differing edge weights should influence the length of the edge. Therefore, the layout has to incorporate the variability of the weights.

**FIGURE 9:**
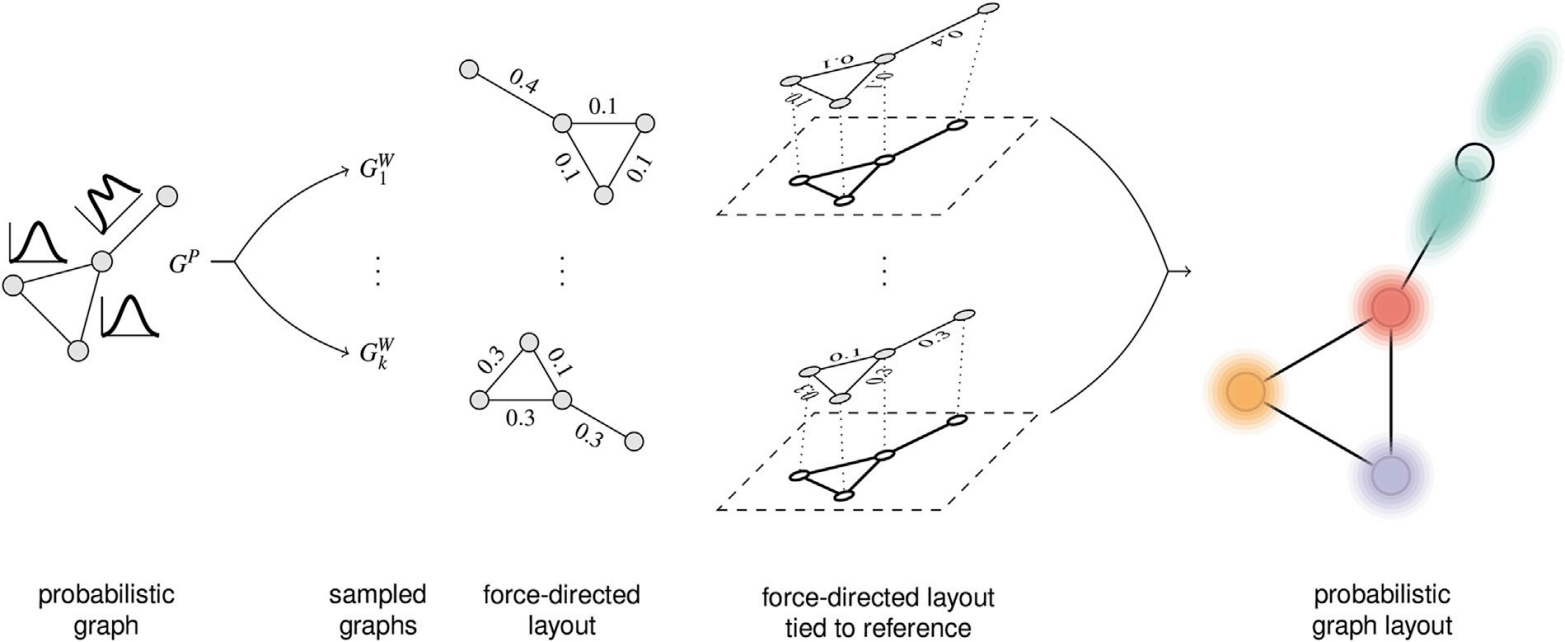
Processing steps for sampling-oriented node-link visualization of uncertain graph data. Image: ^©^ 2017 IEEE. Reprinted, with permission, from Schulz et al. (2017).

A probabilistic graph layout (Schulz et al., 2017) achieves uncertainty visualization by showing distributions implicitly *via* overlay. Figure 9 illustrates the processing steps. First, we need a model of the probabilistic graph. Here, one has to consider whether there are dependencies between the probability density functions for the weights on the different edges. With this uncertainty model, we can then draw samples: these samples are complete graphs with edge weights, albeit each weight is now a fixed value that comes from drawing the sample. The next step produces a graph layout independently for each of the graph samples, here *via* a force-directed graph layout.

As already discussed in Section 4.2, registration or alignment is needed if the individual visualizations do not fit together. This is the case with many graph layout results. Therefore, we need an alignment step, here implemented by tying the individual layouts to a reference layout. In other words, an appropriate layout is a key component in this kind of uncertainty visualization.

The final step renders the overlay of the individual graph visualizations. The basic idea is to perform blending of the individual images. However, this approach would lead to problems caused by visual clutter. Therefore, a combination of splatting nodes, curve bundling for the edges, and adapted node coloring and clustering is used.

**FIGURE 10:**
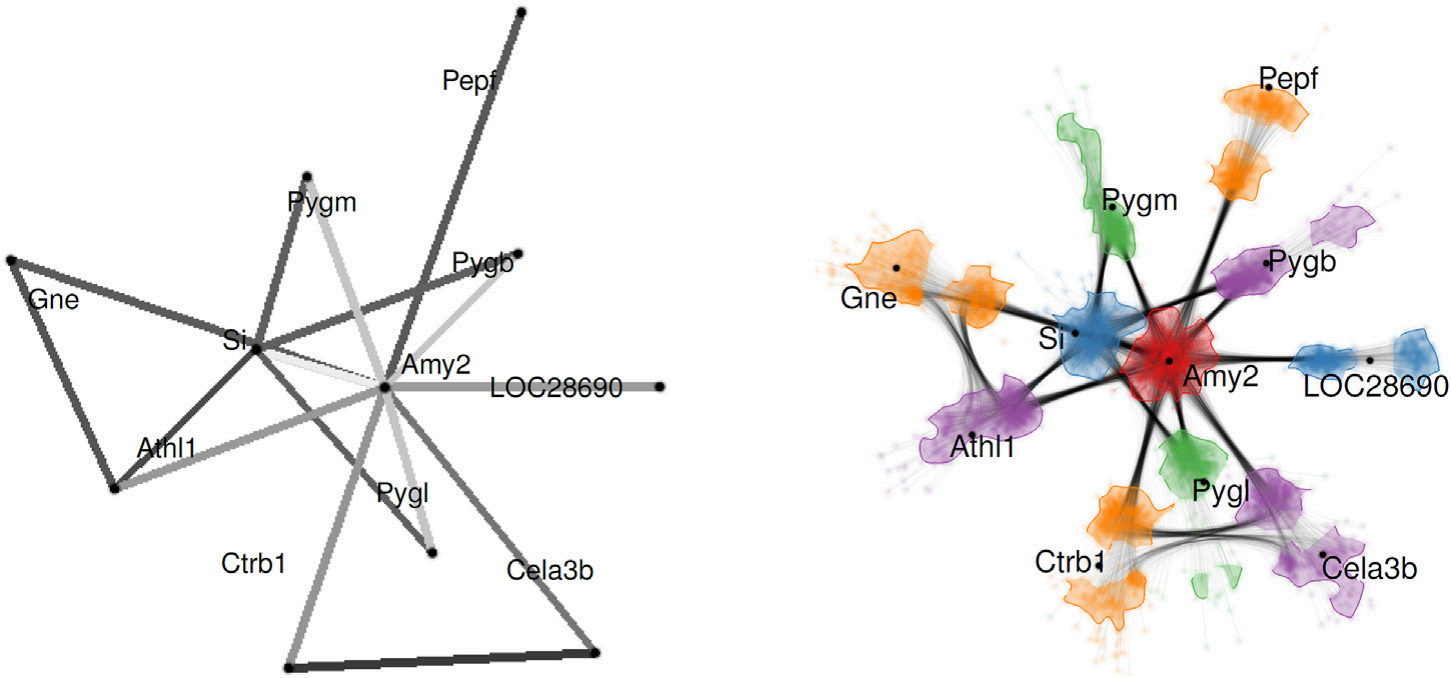
Probabilistic graph layout for visualizing protein–protein interactions for pancreatic alpha-amylase (Amy2). The left image shows the expected (average) graph, i.e., traditional non-uncertainty visualization. The right image shows the uncertainty visualization. Image: ^©^ 2017 IEEE. Reprinted, with permission, from Schulz et al. (2017).

Figure 10 shows an example of probabilistic graph visualization for protein–protein interactions. The edge weights are derived from scores computed from data from the STRING database.^4^ The comparison between the traditional visualization without uncertainty (left image in Figure 10) and the one that incorporates uncertainty (right image in Figure 10) demonstrates varying levels of (un)-certainty associated with the different interactions.

This example is based on an overlay resulting in a static image. By exchanging the last part of the processing pipeline, one could also use small multiple or animation to show the individual graph visualizations coming from the sampling process. For example, Zhang et al. (2022) present and discuss a method based on animation.

**FIGURE 11:**
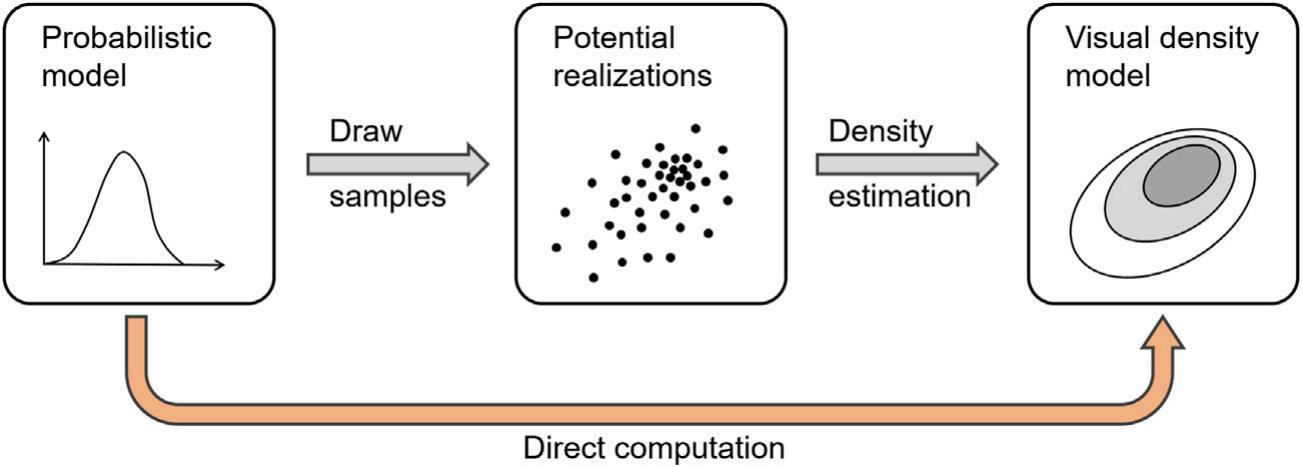
Process of sampling and density estimation for the implicit visualization of distributions.

The sampling approach essentially reduces the problem of uncertainty visualization to the visualization of many individual samples. Figure 11 illustrates the process.

We start with the uncertainty model in the form of probability density functions or similar probabilistic descriptions. From these, points—in a potentially abstract and complex space—are produced by sampling (e.g., Monte-Carlo random sampling, quasi-Monte-Carlo sampling, etc.) and mapped to intermediate images by applying regular non-uncertainty-oriented visualization. In the last step, the images are overlaid to generate the final visualization. As in the example of probabilistic graph visualization, this last step aims to generate a density representation (here, of nodes and edges), e.g., by employing kernel density estimation. Therefore, the process essentially performs a discretization into points and then a reconstruction of a density field, i.e., a numerical approximation with several potential sources for errors and required parameter choices.

Ideally, we would avoid the construction of in-between samples and, instead, directly go from the probability description of the data to the density model of the visual output. This can be readily done when there is no registration needed, such as for typical cases of scientific visualization with given spatial embedding. For example, the probability where an isosurface cuts through the volume can then be mapped to density, which can be rendered by color-coding (Pöthkow and Hege, 2011). However, when the visual mapping implies more complex transformations, the density computation becomes more difficult. For certain scenarios of multidimensional data, there are techniques that construct density plots for parallel coordinates and scatterplots (Bachthaler and Weiskopf, 2008; Heinrich and Weiskopf, 2009; Heinrich et al., 2011) that carry over to respective uncertainty plots (Zheng and Sadlo, 2021). However, developing similar techniques for other advanced examples of uncertainty visualization remains a largely unsolved problem so far.

## 6 Discussion

We have surveyed concepts, strategies, and methods for uncertainty visualization—mostly from the perspective of visualization research. This section discusses general observations, open questions, and directions for future research. In addition, we link this discussion to recommendations geared toward use in applications of biological data visualization.

### 6.1 Open Questions and Future Directions in Visualization Research

We have seen that there has been quite some progress in uncertainty visualization, leading to a large variety of available techniques. However, we have also discussed that uncertainty visualization is challenging due to the difficult, yet relevant interplay of many different components in the visualization process. Therefore, there are a number of directions for future research.

*Layout is key to advanced visual mappings.* One issue with integrating uncertainty information in an already complex visualization is the lack of space, for example, to place glyphs, integrate range representations, use waviness or sketchiness of larger visual marks, etc. Here, the layout process essentially needs to balance the different and conflicting requirements from showing complex data and its uncertainties. Visualization space is a scarce resource in this respect. The example of probabilistic graph visualization exhibits another layout problem: the one of aligning or registering individual visualization images. Therefore, future progress in the visual mapping of uncertainty is related to developing appropriate layout methods that optimize for potentially conflicting goals.

*Perception, cognition, and evaluation.* Understanding how we perceive visualization and reason with it is a central problem in visualization in general; and this problem is even harder when we include uncertainty. Therefore, this topic will continue to play a highly relevant role in uncertainty visualization, and it is tightly connected to ways of how uncertainty visualization is evaluated, e.g., from the user perspective.

*Uncertainty visualization literacy.* There is the general issue of visualization literacy, i.e., dealing with how people can generate and read visualizations. With the progress in uncertainty visualization techniques comes the opportunity of working on improving respective literacy. Due to the difficulties that users have with many visual representations of uncertainty, there is a great potential from the interplay between improving visualization techniques and teaching skillsets.

*Interacting with uncertainty visualization.* We have touched on some examples of interaction techniques geared toward the process of uncertainty visualization. However, this topic is largely untapped so far. We see great potential for future research on interaction methods that will have to include the perceptual and cognitive aspects discussed above.

*Integration with machine learning and explainable AI.* The major trend toward including machine learning also manifests itself in uncertainty visualization. Here, the special interest is in assessing and visually communicating the uncertainty associated with automatic data analysis and machine learning, which also links to visualization as a means to support explainable artificial intelligence (AI).

*Frameworks and software integration.* A message from the consideration of the complete visualization pipeline is: it is not sufficient to just look at stages of the pipeline separately. For example, it is not enough to only consider visual mappings of uncertainty. Instead, there is a need for frameworks that provide a unified perspective. There is already some work on frameworks and integration (e.g., Correa et al. (2009), Wu et al. (2012), and Sacha et al. (2016)), but with the progress coming from the other topics listed above, the frameworks will need to be adapted and extended. In particular, there is the challenge of including the user in the combined process of human–machine visual data analysis. A practical problem is the lack of uncertainty visualization techniques in many existing software systems. Available implementations of uncertainty visualization are often restricted to individual and separate research prototypes. Therefore, there is the need for extended software systems supporting uncertainty visualization.

### 6.2 Recommendations

The lack of widespread implementations of uncertainty visualization is one issue that makes it hard to include it in applications of biological data visualization. Still, there are opportunities for practical impact of uncertainty visualization on bioinformatics applications. Some of the following recommendations might facilitate the integration of uncertainty visualization in such applications.

*Think about data modeling and the context of the visualization process.* An important early step is to understand the data and uncertainty model, which naturally has to be deeply rooted in the application at hand. The next step is to consider the tasks that should be solved with visualization and how they might be affected by data uncertainty. To this end, interdependencies between the components for data acquisition, processing, and visualization should be taken into account, including propagation of uncertainty. Here, rough estimates or models might be sufficient for a coarse description of the interdependencies, and these might be done completely outside of visualization software systems.

*Focus on main players for uncertainty.* Although we argued for the importance of considering the whole visualization process, it is clear that not all stages are equally important for each application. Instead, it is better to focus the attention on the main sources and effects of uncertainty. Then, only these parts of the whole process might have to be extended from regular non-uncertainty processing to an uncertainty-aware counterpart. This approach can reduce the effort substantially, especially when there is no comprehensive uncertainty visualization system available.

*Choose appropriate visualization techniques.* In general, visualizations should be chosen to match data characteristics, tasks, and intended audience. Usually, there is not a single-best method. This statement is especially true for uncertainty visualization. For example, existing multivariate data visualization might be enough for your own internal processes of data analysis, but not for effective communication to a broader outside audience. The choice of visualization technique might also be related to the availability of implementations (or lack thereof). Some visual mappings are easier to integrate into existing non-uncertainty-oriented visualization techniques than others. For example, per-pixel visual variables like color or others tend to be easy to integrate into existing non-uncertainty-oriented visualization systems; it might be as simple as modifying the color map or extending multivariate visualization in parallel coordinates with additional data axes. Other uncertainty mappings require much more work, for example, when there is a serious impact on the layout or when comprehensive systems have to be changed for a full visual analytics framework for uncertainty. Such efforts in modifying or implementing visualization techniques should play a role in choosing appropriate techniques.

*Need for integration in existing software.* In general, there is a lack of comprehensive uncertainty support in existing visualization software in many bioinformatics applications. Therefore, some community effort could help with including more of the uncertainty-aware stages of the visualization pipeline.

*Uncertainty awareness.* Due to the complexity of uncertainty visualization, there might not be a single and comprehensive solution. Instead, the main goal of this paper is increased awareness of issues that come with uncertainty in visualization.

## Footnotes

1 D. Weiskopf: Uncertainty Visualization. Keynote presentation at the 11th International Meeting on Visualising Biological Data (VIZBI 2021)

2
https://namastevis.github.io/uncertaintyVizBrowser/

3 BELIV: Evaluation and Beyond – Methodological Approaches for Visualization, https://beliv-workshop.github.io

4
https://string-db.org/
